# KampoDB, database of predicted targets and functional annotations of natural medicines

**DOI:** 10.1038/s41598-018-29516-1

**Published:** 2018-07-25

**Authors:** Ryusuke Sawada, Michio Iwata, Masahito Umezaki, Yoshihiko Usui, Toshikazu Kobayashi, Takaki Kubono, Shusaku Hayashi, Makoto Kadowaki, Yoshihiro Yamanishi

**Affiliations:** 1 Medical Institute of Bioregulation, Kyushu University, 3-1-1 Maidashi, Higashi-ku, Fukuoka, Fukuoka, 812-8582 Japan; 2 Department of Bioscience and Bioinformatics, Faculty of Computer Science and Systems Engineering, Kyushu Institute of Technology, 680-4 Kawazu, Iizuka, Fukuoka 820-8502 Japan; 3 Division of Chemo-Bioinformatics, Institute of Natural Medicine, University of Toyama, Toyama, 930-0194 Japan; 4 Division of Gastrointestinal Pathophysiology, Institute of Natural Medicine, University of Toyama, Toyama, 930-0194 Japan; 5 PRESTO, Japan Science and Technology Agency, Kawaguchi, Saitama 332-0012 Japan

## Abstract

Natural medicines (i.e., herbal medicines, traditional formulas) are useful for treatment of multifactorial and chronic diseases. Here, we present KampoDB (http://wakanmoview.inm.u-toyama.ac.jp/kampo/), a novel platform for the analysis of natural medicines, which provides various useful scientific resources on Japanese traditional formulas Kampo medicines, constituent herbal drugs, constituent compounds, and target proteins of these constituent compounds. Potential target proteins of these constituent compounds were predicted by docking simulations and machine learning methods based on large-scale omics data (e.g., genome, proteome, metabolome, interactome). The current version of KampoDB contains 42 Kampo medicines, 54 crude drugs, 1230 constituent compounds, 460 known target proteins, and 1369 potential target proteins, and has functional annotations for biological pathways and molecular functions. KampoDB is useful for mode-of-action analysis of natural medicines and prediction of new indications for a wide range of diseases.

## Introduction

Traditional medicines are used clinically in many areas of the world, including in Japan (Kampo), China, Korea, India (Ayurveda) and Perso-Arabic countries (Yunani). Traditional medicines usually comprise mixtures of the crude extracts from several medicinal herbs, each of which contains multiple components. The World Health Organization took the initiative to promote the globalization of traditional medicine in 1972 by founding a Division of Traditional Medicine. Approximately 45 years later, traditional medicines are widely available and are commonly used in many parts of the world. Recently, there has been a dramatic worldwide increase in the number of patients suffering from complex diseases, such as lifestyle-related diseases, cardiovascular diseases, diabetes, and immune-mediated diseases. It can be difficult to cure these complex diseases effectively with Western medicines by using the “one disease, one target, one drug” approach, and there are growing expectations toward the “one disease, multiple targets, multiple drugs” approach with multi-effective drugs such as traditional medicines used in combination therapies with Western medicines.

Kampo medicine originated from ancient Chinese medicine but evolved independently over a long period of time (more than 1500 years) to become a style individual to Japan. Kampo formulas often differ from Chinese or Korean traditional formulas, although many of the same medicinal herbs are used for traditional medicines across eastern Asian countries. Kampo medicines are decoctions or dry powders that include pharmaceutical active ingredients extracted by boiling from a mixture of naturally derived medicinal herbs. They are generally factory-produced by pharmaceutical companies in Japan and provided in a ready-to-use form. To assure the quality of Kampo products, the Japanese Ministry of Health, Labour and Welfare published their “Guideline on Data Requirements for Ethical Kampo Formulation” in 1985, resulting in Kampo medicines becoming standardized with respect to the quality and quantity of their ingredients. The Ministry maintains oversight of Kampo medicines. Japanese traditional formulas Kampo medicines are prescribed in hospitals in Japan as either monotherapy or harmoniously combined therapy with standard western therapy and >80% of medical doctors prescribe Kampo medicines in Japan^1^. Thus, Kampo medicines are established as a pivotal part of mainstream medicine in Japan and the cost of Kampo medicines is covered by the National Health Insurance. On the other hand, in the United States, National Institutes of Health (NIH) now supports clinical and basic research on the traditional medicine. In recent years, NIH support and the US Food and Drug Administration (FDA) guideline on investigating botanical drug products, including complex formulas containing many constituents, has fostered the development of botanical drugs in the United States^1,2^. Presently, randomized, double-blind, placebo-controlled clinical trials of some Kampo medicines (e.g., Daikenchuto for bowel diseases) are underway for phase II or phase III studies for FDA approval in the United States.

**Figure 1:**
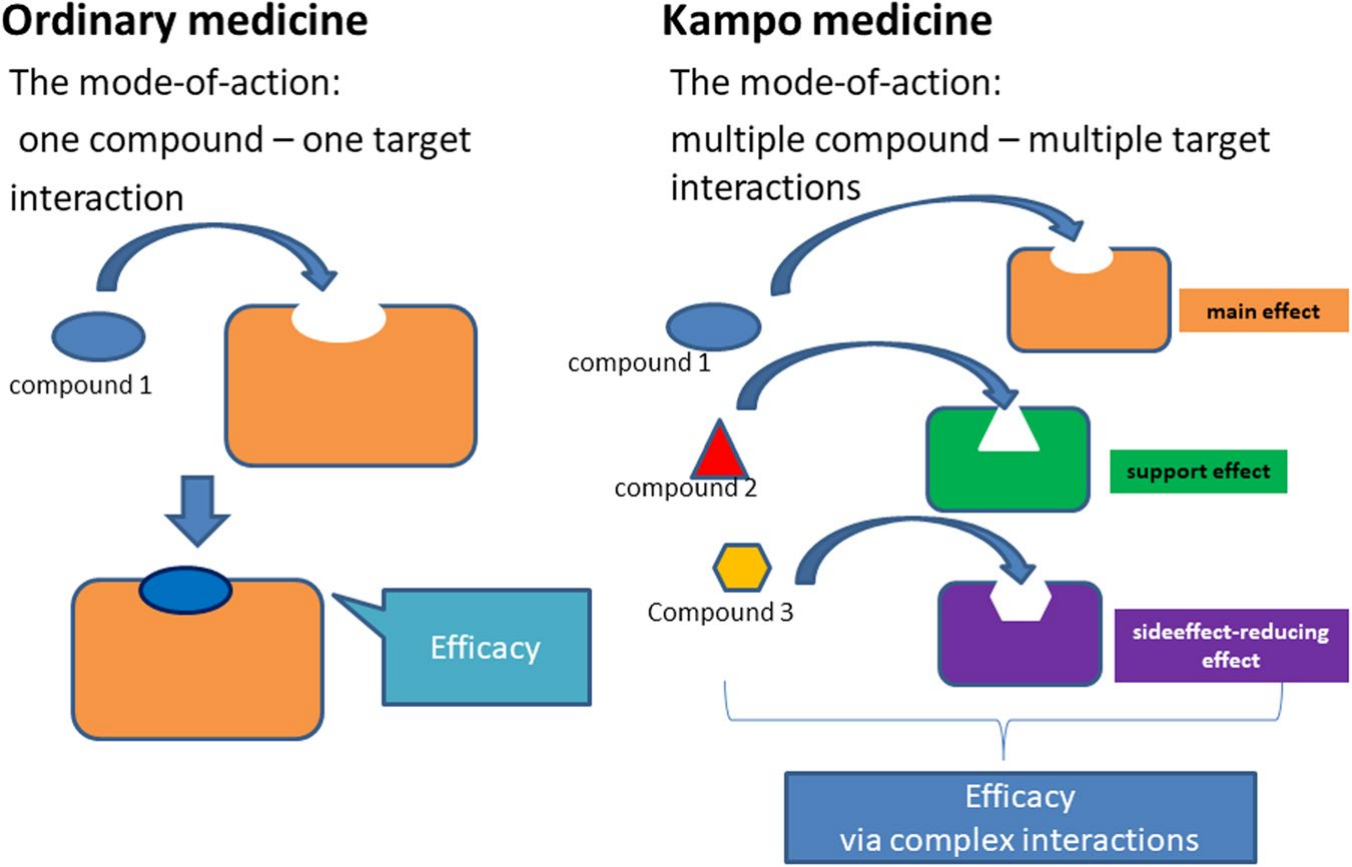
An illustration of the difference of the mode-of-action between ordinary medicines and Kampo medicines. In ordinary medicines, the efficacies stem from one compound–one target interaction. On the other hand, in Kampo medicines, the efficacies stem from multiple compound–multiple target interactions.

However, the pharmacotherapy with Kampo medicines greatly depends on the empirical knowledge of medical doctors in practice, and there is insufficient scientific evidence explaining the underlying molecular mechanisms of Kampo medicines. The mechanisms of Kampo medicines are different from those of ordinary medicines. The efficacies of Kampo medicines stem from multiple compound–multiple target interactions. Figure 1 shows an illustration of the difference of the mode-of-action between ordinary medicines and Kampo medicines. It is, therefore, indispensable to establish fundamental technologies to comprehensively analyze the underlying mechanisms of every pharmacological action of multicomponent Kampo medicines in the human body as a complex system.

In recent biomedical science, clinical and molecular data for Kampo medicine-based pharmacotherapy have been accumulated, and a variety of omics data are becoming available in the genome, transcriptome, proteome, metabolome, phenome, and diseasome. These “big data” are useful resources for mode-of-action analysis of Kampo medicines; thus, there is a strong need to develop databases and associated tools for Kampo medicines. Many databases for Western medicines exist (e.g., DrugBank^3^, KEGG DRUG^4^, Matador^5^, SuperTarget^5^, ChEMBL^6^, Therapeutic Target Database^7^, BindingDB^8^, PubChem^9^, Comparative Toxicogenomics Database^10^). However, there is no integrated database of Kampo medicine-related chemical and biological data, and clinical research data and clinical findings. There is a wiki-system database of Kampo medicines and crude drugs^11^, but it is mainly Kampo medicine-related pharmacognostical and chemical database and thereby cannot help to understand the mode-of-actions and further clinical applications of Kampo medicines.

Here, we present KampoDB (http://wakanmoview.inm.u-toyama.ac.jp/kampo/), a novel platform for the analysis of natural medicines, which provides various useful scientific resources on Kampo medicines, constituent herbal medicines, constituent compounds, and target proteins of these constituent compounds. Potential target proteins of these constituent compounds were predicted by docking simulations and machine learning methods based on large-scale omics data (e.g., genome, proteome, metabolome, interactome). Therefore, KampoDB is useful for understanding the mode-of-action of natural medicines in terms of biological pathways and molecular functions of target proteins, which can lead to new indications for a wide range of diseases. The present study aims to elucidate the underlying mechanisms of Kampo medicines, while predicting their target proteins and new indications, thereby repositioning Kampo medicines for their extensive application in clinical practice, with a view toward using them more effectively in clinical practice.

## Results

### Data collection

The current version of KampoDB contains 42 Kampo medicines, 54 crude drugs, 1230 constituent compounds, 460 known target proteins, and 1369 potential target proteins and has functional annotations for biological pathways and molecular functions. The molecular information on natural medicines in KampoDB was collected and digitized from scientific literature, molecular databases, and clinical reports. We collected the relationships between Kampo drugs and crude drugs (and also below layers) from the Traditional Medical & Pharmaceutical Database of the Institute of Natural Medicine, University of Toyama (http://wakankensaku.inm.u-toyama.ac.jp/). As the information was provided in Japanese, we translated it to English. The correlation between Kampo drugs and crude drugs was not based on the computational predictions. The mode-of-action was elucidated by applying the state-of-the-art computational methods (see the METHODS section for more details). KampoDB is compatible with other molecular biology databases (e.g., KEGG^12^, ChEMBL^6^, UniProt^13^, KNApSAcK^14^) by using the same identifiers (compound IDs, protein IDs, disease IDs).

### Inputs and outputs

**Figure 2:**
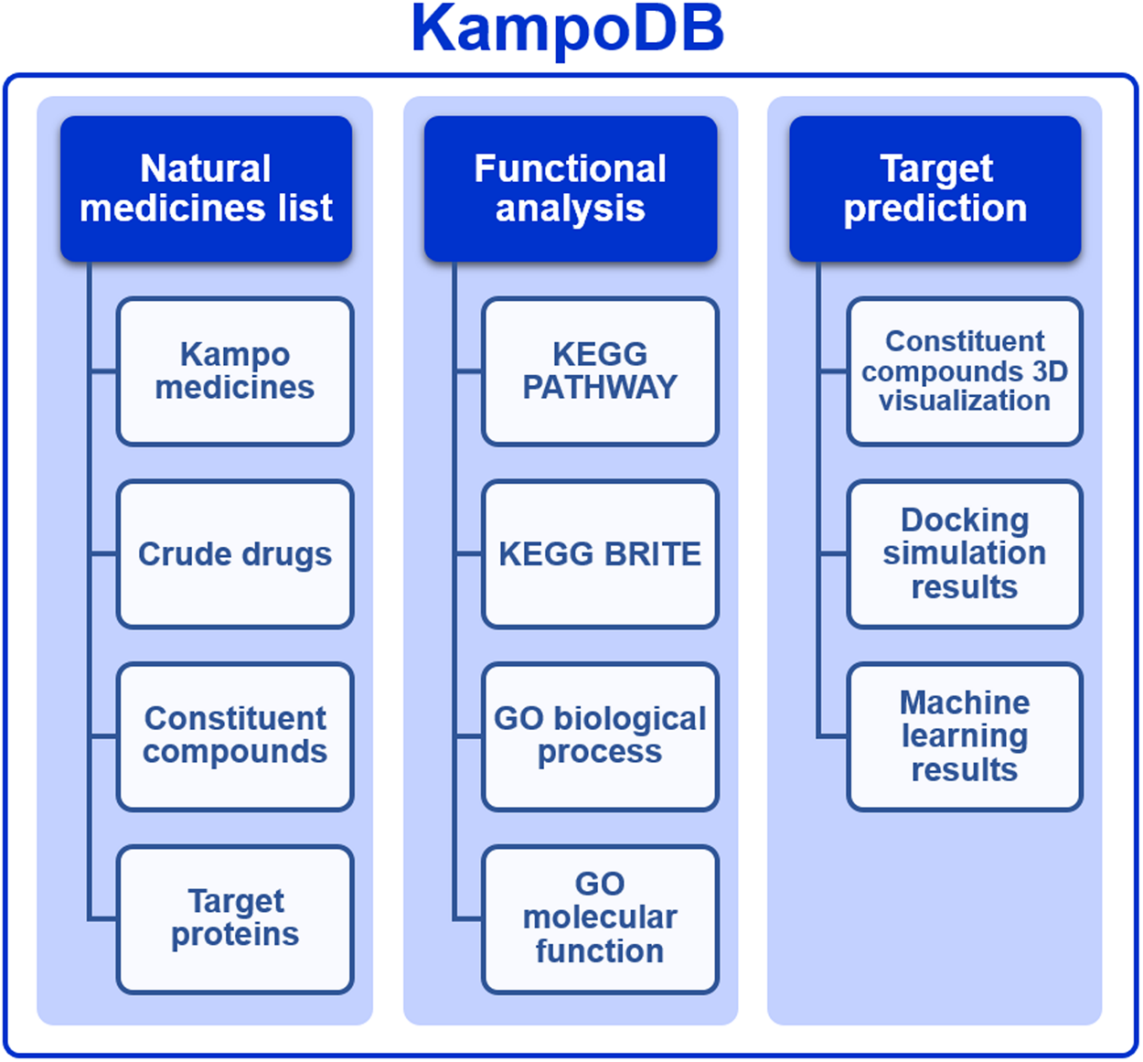
A diagrammatic representation of KampoDB that consists of three components: (1) natural medicines list, (2) functional analysis, and (3) target prediction.

KampoDB consists of three components: 1) natural medicines list, 2) functional analysis, and 3) target prediction. Figure 2 shows a diagrammatic representation of KampoDB. All of the resources are accessible via the website (http://wakanmoview.inm.u-toyama.ac.jp/kampo/).

In the “Natural medicines list” component, a user can input a natural medicine name (e.g., “kakkonto”) as a query. Kakkonto is one of the most frequently used Kampo medicines in Japan, because it is a highly effective and safe medicine against the common cold^15^, influenza^16^ and allergic rhinitis^17^ either as sole therapy or in combination with modern Western medicines. Kakkonto is composed of seven Japanese Pharmacopoeia standard medicinal herbs: *Puerariae Radix*, *Cinnamomi Cortex*, *Zizyphi Fructus*, *Paeoniae Radix*, *Ephedrae Herba*, *Zingiberis Rhizoma* and *Glycyrrhizae Radix*. The main bioactive compound in kakkonto is thought to be puerarin, which is an isoflavonoid derived from *Puerariae Radix* that exhibits many pharmacological properties, including such as anti-inflammation, vasodilation, neuroprotection, antioxidant and anticancer effects (Supplementary Fig. 1)^18^. Clicking on the search button, the user can obtain the corresponding information on Kampo medicines, crude drugs, constituent compounds and target proteins. Note that compound IDs correspond to KNApSAcK IDs^14^, and protein IDs correspond to KEGG GENES IDs^12^. The user can see a global classification of Kampo medicines, crude drugs, constituent compounds, and target proteins in a hierarchical manner (Kampo medicines on the 1st layer; crude drugs on the 2nd layer; constituent compounds on the 3rd layer; target proteins on the 4th layer). Note that each Kampo medicine consists of multiple crude drugs, each crude drug consists of multiple compounds, and each constituent compound is supposed to interact with its target proteins. If the proteins are therapeutic targets of diseases, the corresponding diseases are shown.

In the “Functional analysis” component, the user can input natural medicine names. The output is the summary of the mode-of-action analysis of the corresponding natural medicines, which provides molecular function annotations of target proteins (e.g., molecular functions in Gene Ontology^19^) and biological pathway annotations (e.g., biological pathways in KEGG PATHWAY^12^). A visualization of the results at different layer levels enables the user to see the mode-of-action information in a hierarchical manner within a natural medicine classification. The user can select one option from the following four categories and click on the corresponding button: (1) Pathway: biological pathways in the KEGG PATHWAY, (2) Brite: protein classifications in KEGG BRITE, (3) Process: biological process terms in GO, and (4) Function: molecular function terms in GO. For example, in the case of “Pathway”, the output is the list of pathway names with high enrichment ratio scores and low p-values (See the METHODS section for more details).

In the “Target prediction” component, the user can see the results of newly predicted target proteins of major constituent compounds by performing docking simulations and machine learning techniques. The user can select a query compound by clicking on a compound name of interest in the list of the constituent compounds that are defined as standard compounds in the Japanese pharmacopoeia (see the METHODS section for more details). The outputs are the list of predicted human proteins for the query compound and associated information. In the docking simulation method, docking was performed for the constituent compounds with each human protein 3D structure. In the machine learning method, supervised classification with compound chemical structure similarity was performed for each human protein (see the METHODS section for more details).

### Possible applications

**Figure 3:**
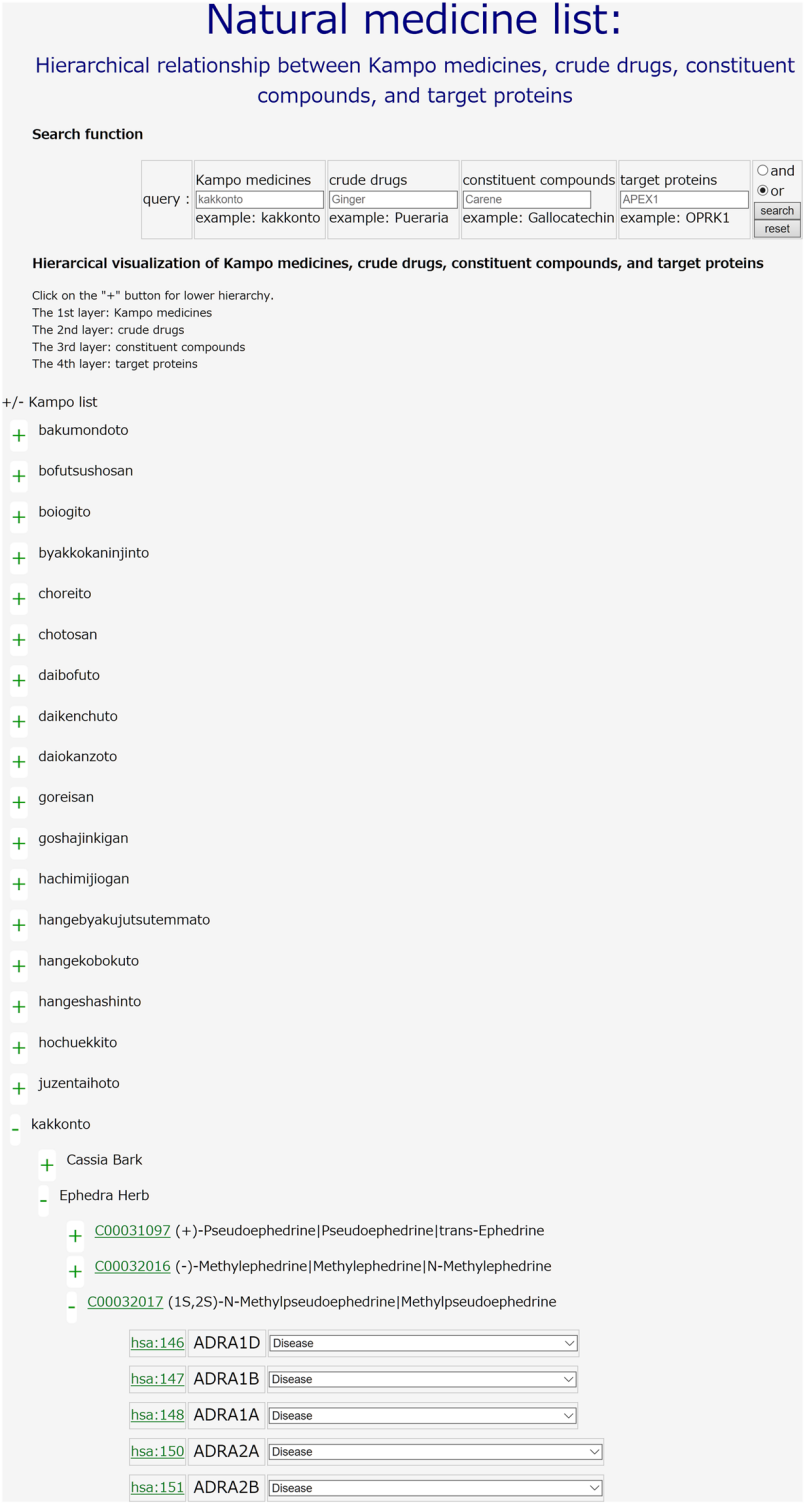
An example of the output page of the query “kakkonto” (an example of Kampo medicines) as an input in the “Natural medicines list” component. The 1st layer shows the Kampo medicine query (“kakkonto” in this case). The 2nd layer shows the crude drugs (e.g., “Ephedra herb”) that form the Kampo medicine (“kakkonto” in this case). The 3rd layer shows the constituent compounds (e.g., “Methylephedrine”) that form the crude drug (“Ephedra herb” in this case). The 4th layer shows the target proteins (e.g., “ADRA1D”) that are known to interact with the constituent compound (“Methylephedrine” in this case).

An application of the “Natural medicines list” component in KampoDB is to view a hierarchical classification of natural medicines. Figure 3 shows an example of the output page of the query “kakkonto” (an example of Kampo medicines) as an input. The 2nd and 3rd layers show the crude drugs (e.g., “Ephedra herb”) constituting the Kampo medicine (“kakkonto” in this case) and the compounds (e.g., “Methylephedrine”) constituting the crude drug (“Ephedra herb” in this case), respectively. The 4th layer shows the target proteins (e.g., “ADRA1D”) that are known to interact with the constituent compound (“Methylephedrine” in this case). The output enables the user to investigate the hierarchical relationship between Kampo medicines, crude drugs, constituent compounds and target proteins.

**Figure 4:**
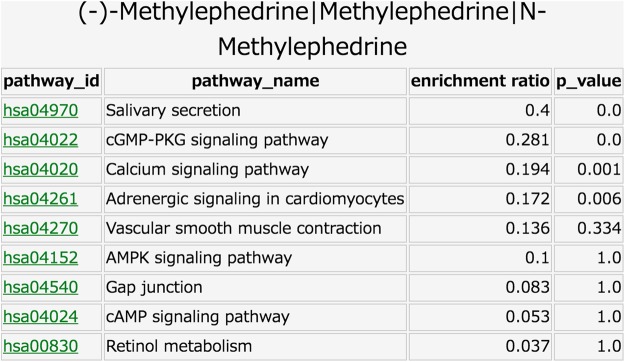
An example of the output page of the query “Methylephedrine” (an example of constituent compounds of “kakkonto”) as an input in the “Functional analysis” component. The 1st column shows the pathway ID in KEGG, the 2nd column shows the pathway name, the 3rd column shows the enrichment ratio, and the 4th column shows the p-value for a hypergeometic test.

An application of the “Functional analysis” component in KampoDB is to perform the mode-of-action analysis of natural medicines in terms of biological pathways and molecular ontologies. Figure 4 shows an example of the output page of the query “Methylephedrine” (an example of constituent compounds of “kakkonto”) as an input in the “Functional analysis” page. In the case of pathway enrichment analysis, biological pathways with high enrichment ratios and low p-values can be thought of as candidates for the associated pathways. For example, the “cGMP-PKG signaling pathway”, “Calcium signaling pathway”, and “Adrenergic signaling in cardiomyocytes” were detected as the pathways associated with the term “Methylephedrine.” This is a reasonable result because target proteins of methylephedrine (e.g., ADRA1D, ADRA1B, ADRA1A) are known to be involved in the adrenergic signaling process^4^.

**Figure 5:**
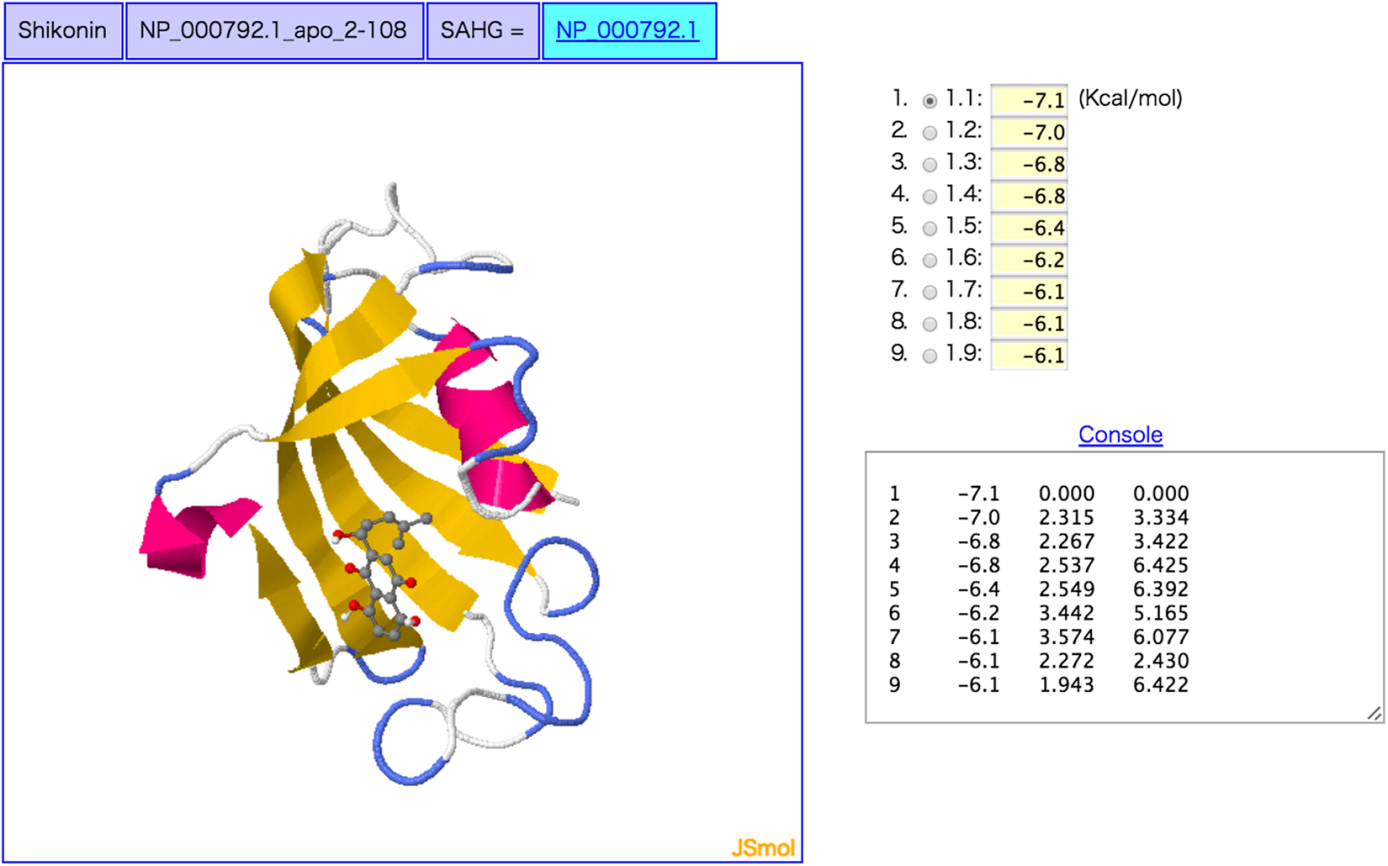
An example of the output page of the query “shikonin” (a constituent compound of “*Lithospermum erythrorhizon*”) with the docking simulation option in the “Target prediction” component.

An application of the “Target prediction” component in KampoDB is to predict unknown target proteins of the constituent compounds of natural medicines. Figure 5 shows an example of the output page of the query “shikonin” (a constituent compound of “*Lithospermum erythrorhizon*”) with the docking simulation option in the “Target prediction” component. The left panel in Fig. 5 shows the binding form of the predicted interaction between shikonin with FK506-binding protein (FKBP). The graphical picture enables the user to investigate the ligand binding sites on the protein 3D structure. The validity of the shikonin-FKBP interaction and its pharmacological effects were experimentally confirmed in a previous work^20^.

**Figure 6:**
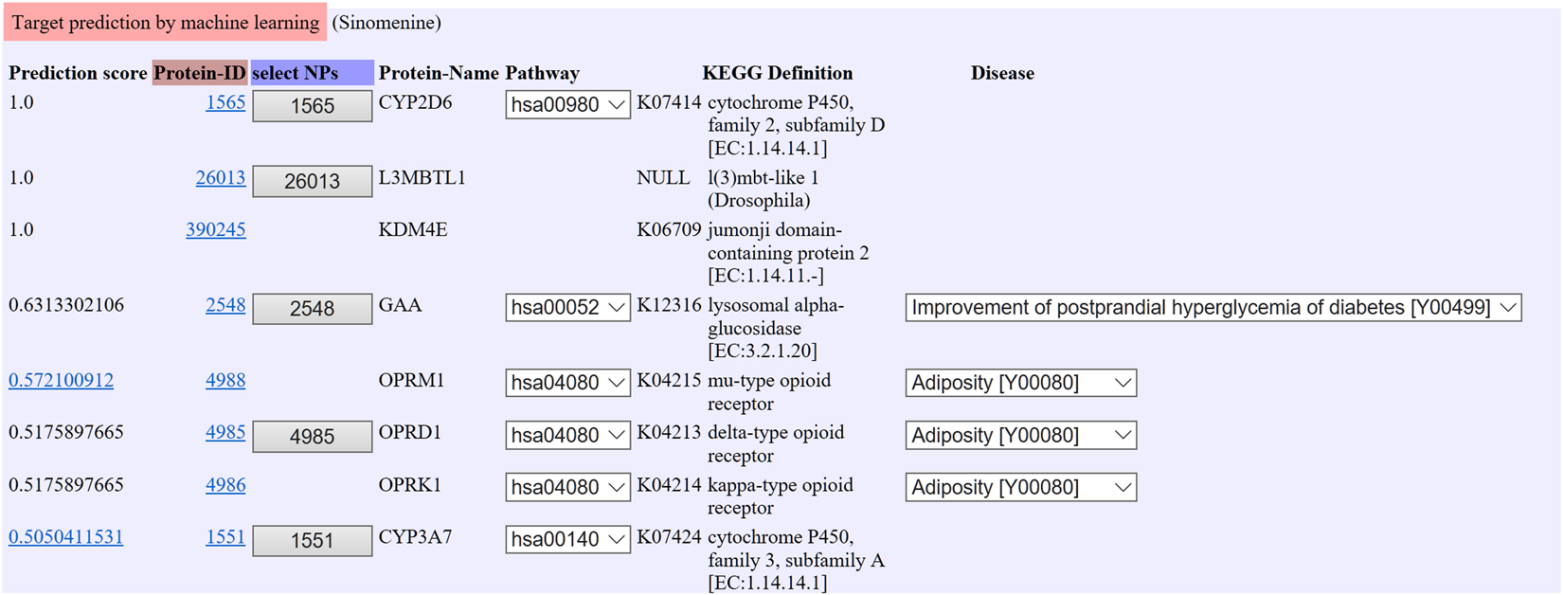
An example of the output page of the query “Sinomenine” (a constituent compound of “boiogito”) with the machine learning option in the “Target prediction” component.

Figure 6 shows an example of the output page of the query “Sinomenine” (a constituent compound of “boiogito”: see red rectangle in Supplementary Fig. 2) with the machine learning option in the “Target prediction” component. Boiogito is prescribed as a Kampo remedy for arthritis, nephrosis, edema, hyperhidrosis and obesity. Boiogito is composed of six Japanese Pharmacopoeia standard medicinal herbs: *Sinomeni Caulis et Rhizoma*, *Astragali Radix*, *Atractylodis Lanceae Rhizoma*, *Zizyphi Fructus*, *Glycyrrhizae Radix* and *Zingiberis Rhizoma*. Sinomenine, an ingredient extracted from the *Sinomenium Stem*, exerts anti-inflammatory effects through inhibiting lymphocyte proliferation^21^, and decreasing eicosanoid synthesis and nitric oxide production^22^. Furthermore, sinomenine ameliorates experimental arthritis in an animal model^23^. The list of target candidate proteins and the associated information (e.g., molecular functions, biological pathways, applicable diseases) are shown with a ranking from the highest prediction score. Examples of predicted applicable diseases of sinomenine are adiposity and type II diabetes mellitus, implying that sinomenine is effective for treatment of adiposity and type II diabetes mellitus based on the target proteins: GAA, OPRM1, OPRD1, OPRK1. These observations are reasonable, because Kampo medicine “boiogito” that includes sinomenine as a constituent compound is known to be useful for adiposity. These results also suggest that GAA, OPRM1, OPRD1, and OPRK1 may play key roles in the pharmacological action of “boiogito”. This is how the method can be used for the mode-of-action analysis of Kampo medicines.

### A case study

As a case study, we show here how KampoDB could be used with daikenchuto, one of the most frequently used Kampo medicines in Japan. Daikenchuto is beneficial for postoperative complications such as ileus and abdominal bloating. Although the mechanisms of daikenchuto are not fully understood, it has been reported that daikenchuto ameliorates these intestinal motility disorders via the release of serotonin and suppress the inflammation via the inhibition of cyclooxygenase-2 activity^24,25^.

When “daikenchuto” was entered as a query in KampoDB, the “Functional analysis” component predicted “serotonergic synapse” and “arachidonic acid metabolism” as the associated pathways. It also predicted “Wnt signaling pathway”, “T cell receptor signaling pathway”, and “TNF signaling pathway” as candidates for target pathways associated with the mechanisms of daikenchuto. This suggests that daikenchuto derives its anti-inflammatory activity via arachidonic acid metabolism^26^ and several other pathways. “T cell receptor signaling pathway” and “TNF signaling pathway” have been supported by previous reports^27,28^ as the underlying mechanisms of daikenchuto. However, to the best of our knowledge, there is no report on the role of daikenchuto in the “Wnt signaling pathway”.

We previously showed that daikenchuto markedly alleviated dextran sulfate sodium (DSS)-induced experimental colitis in mice. Ulcerative colitis is a chronic inflammatory bowel disease (IBD) in which patients experience intermittent remission and relapse over decades. The long-term chronic inflammation elevates the risk of colitis-associated cancer (CAC) and can lead to CAC-related death. Therefore, CAC is regarded as the most serious complication of IBD. However, not all medicines effective against experimental colitis are necessarily effective against CAC. Indeed, it has been reported that an agonist for a prostaglandin E2 receptor subtype suppresses DSS-induced colitis and also prevents the development of colorectal carcinogenesis in a murine CAC model, whereas sulfasalazine, a prodrug of 5-aminosalicylic acid with efficacy against DSS-induced colitis, did not prevent colorectal tumor formation in a murine CAC model^29^.

Using KampoDB, “Wnt signaling pathway”, “T cell receptor signaling pathway”, and “TNF signaling pathway” were predicted as candidates for target pathways associated with the underlying mechanisms of daikenchuto that contribute to the development of CAC. In particular, the contribution of the Wnt signaling pathway to the colorectal carcinogenesis is established^30^. Recently, it was reported that the activation of Wnt/β-catenin signaling is essential for the early phase development of IBD-associated colorectal cancer^31,32^. Additionally, Wnt signaling-initiated tumorigenesis has been reported in a murine CAC model^33^.

**Figure 7:**
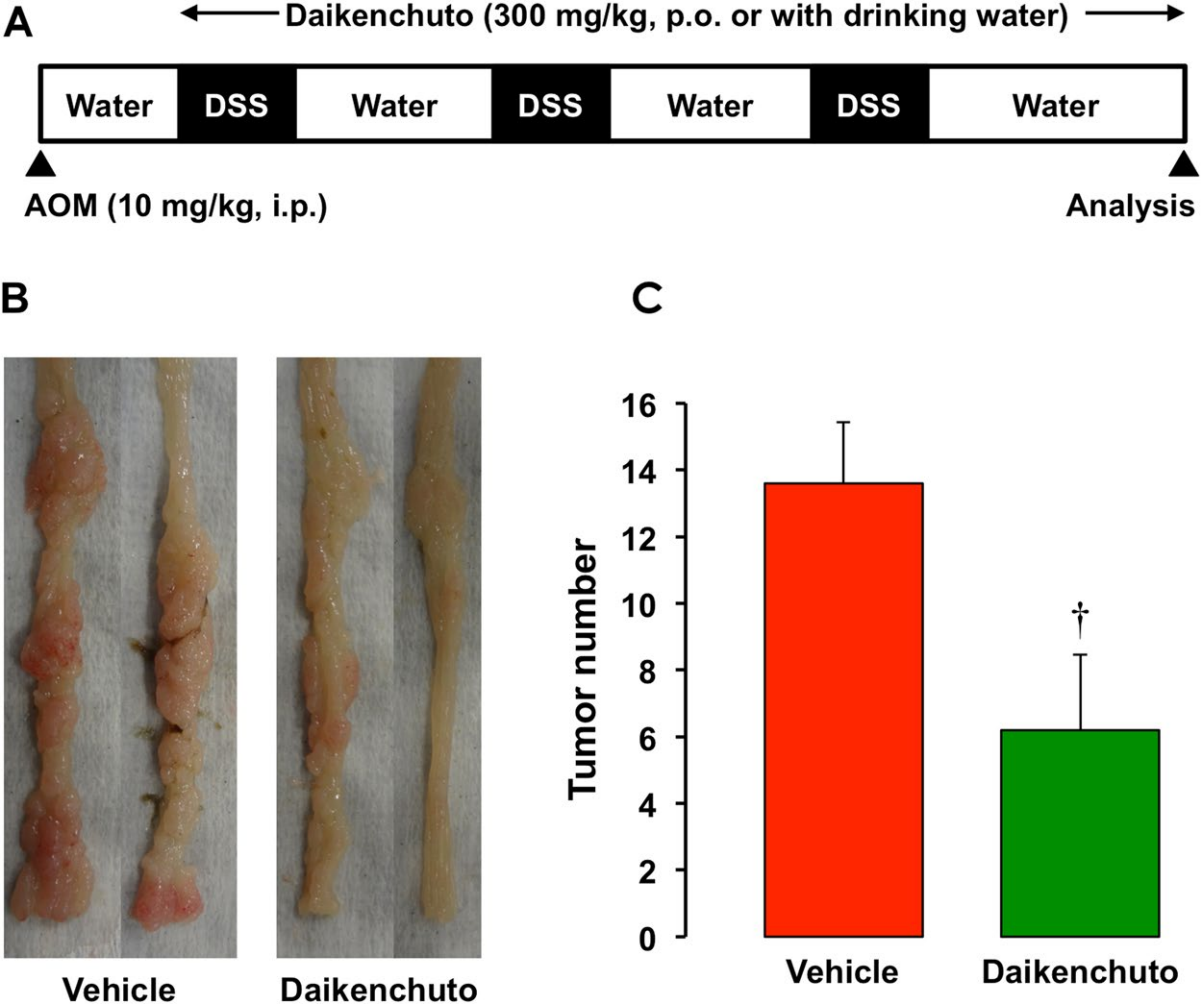
Effect of daikenchuto on the development of colitis-associated cancer (CAC) in mice. CAC was induced in mice by intraperitoneal injection of azoxymethane (AOM) (10 mg/kg) followed by repeated exposure to a 2% dextran sulfate sodium (DSS) in drinking water. Daikenchuto (300 mg/kg) was orally administered during experiment. (**A**) Schematic drawing of the experimental design for the evaluation of daikenchuto in the CAC model. (**B**) Macroscopic changes in the colon. Colons were removed from vehicle- or daikenchuto-treated mice at day 70, and representative results from 5 independent animals are shown. (**C**) The number of tumors. Colons were removed at 70 days to determine the number of macroscopic tumors. The data are presented as means ± SE of 5 mice. ^†^Significant difference from vehicle at p < 0.05.

Taking all together, these findings suggest that daikenchuto attenuates the development of chronic inflammation-associated cancer. It has the potential to be a new therapeutic strategy while repositioning the use of Kampo medicine. While testing this hypothesis, we found that the daikenchuto treatment indeed significantly suppressed the development of chronic colitis-associated colon cancer in a murine experimental model, as shown in Fig. 7.

Daikenchuto comprises three medicinal herbs: *ginseng root*, *processed ginger*, and *Zanthoxylum peel* (Supplementary Fig. 3). KampoDB was able to predict the possibility that the Wnt signaling pathway was a target of *ginseng root* and that the T cell receptor and TNF signaling pathways were underlying mechanisms of the anti-CAC effects of *processed ginger* and *Zanthoxylum peel*. These results suggest that the additive or synergistic actions of constitutive medicinal herbs contribute to the suppressive effect of daikenchuto on the development of CAC. Therefore, KampoDB can be useful for predicting new roles or aspects of traditional medicines, helping to clarify the underlying mechanisms of traditional medicines.

## Discussion

KampoDB is the first platform for the analysis of natural medicines for mode-of-action analysis and repositioning of natural medicines in the world. The primary contribution of this study is to propose computational methods for the mode-of-action analysis and repositioning of Kampo medicines. In this study, we put great efforts on establishing a methodology for the computational prediction of target proteins and new indications of Kampo medicines. We established a useful web service that makes it easier for medical doctors to use Kampo medicines in clinical practice. The methods are expected to be useful for analyzing the complex systems of natural medicines. Thus, the technologies should contribute to innovation in the field of health science.

A related work of this study is a wiki-system of Kampo medicines and crude drugs^11^ and the Kampo section of the KNApSAcK^14^ database that enables group search of medicinal plants, formula search by a medicinal plant, and medicinal plant search by a Kampo formula. However, these existing databases do not provide the information on potential target proteins, target pathways, and applicable diseases. Thus, they cannot help to understand the mode-of-actions and further clinical applications of Kampo medicines and crude drugs.

The performances of the target prediction and indication prediction depend heavily on the data representation of Kampo medicines, crude drugs, constituent compounds, and proteins. In this study, we used chemical structures of the constituent compounds and protein structures, but another approach would be to use other omics data. Recently, compound-induced transcriptome data (e.g., chemical treatment on human cell lines) and genetically-perturbed transcriptome data (e.g., gene knockdown, gene overexpression) have been utilized in various pharmaceutical applications. Similarity, the analysis of gene expression profiles by perturbations with Kampo medicines and crude drugs would be an interesting approach for target prediction and indication prediction. The inclusion of these gene expression data will be one of our important future works.

Traditional medicines have considerable advantages, such as the abundance of clinical experience gained over a long time, the diversity of chemical structures of the constituent compounds, and their biological activity in humans, providing an incomparable source of new drug leads for effective drug development. The results of the present study provided possible concepts and methodologies from traditional medicine that could help the discovery and development of new drugs.

We plan to maintain KampoDB by updating the molecular data on a regular basis and by analyzing the data using more sophisticated computational methods. For the “Natural medicine list” and “Functional analysis” components, we intend to incorporate the latest information from the literature and from other molecular databases. For the docking simulation analysis in the “Target prediction” component, we plan to perform docking simulations for missing compound–protein pairs as soon as the information on protein structures becomes available and to investigate the possibility of using other docking software, such as myPresto. For the machine learning analysis in the “Target prediction” component, we plan to use more sophisticated machine learning methods (e.g., deep learning, support vector machine, and logistic regression) to improve its accuracy in predicting target proteins and the applicable diseases. Currently, the prediction results for applicable diseases are presented at the level of the constituent compounds of the Kampo medicines and crude drugs, but we intend to develop integrative methods to show the prediction results for applicable diseases at the level of Kampo medicines and crude drugs themselves. In Japanese traditional medicines, various kinds of Kampo medicines, crude drugs, and constituent compounds exist. Our KampoDB is just the first version and does not cover all Kampo medicines and diseases. In our future versions, we will add more Kampo medicines, crude drugs, constituent compounds, and diseases.

## Methods

### Chemical structure representation

The chemical structures of constituent compounds were obtained from KNApSAcK^14^ and PubChem^9^ and were represented by their KEGG Chemical Function and Substructures (KCF-S) descriptors^34^. Each compound was coded by a high-dimensional feature vector in which each element indicates the frequency of a feature defined by KEGG Chemical Function Substructures (KCF-S) (i.e., chemical substructures). The number of features was 475,692. We computed chemical structure similarity scores of compounds by using the generalized Jaccard correlation coefficient.

### Compound–protein interactions

Known compound–protein interactions were acquired from public databases: ChEMBL^6^, MATADOR^5^, DrugBank^3^, the Psychoactive Drug Screening Program Ki, KEGG DRUG^4^, the Binding DB^8^, and the Therapeutic Target Database^7^. For the ChEMBL data, we selected only compound–protein interaction pairs that were clearly denoted as active interactions or had binding affinities of <30 μM (e.g., IC_50_), which yielded 1,287,404 compound–protein interactions involving 519,061 compounds and 3,735 proteins. Compounds and proteins included in the chemical–protein interactome data are referred to as interactome compounds and interactome proteins, respectively.

### Constituent compounds

Kampo formulas are recognized as official prescription drug and listed in the Japanese pharmacopoeia. We selected 80 compounds derived from constituent medicinal herbs of Kampo formulas that are most frequently used for the medical treatment in Japan. 80 compounds are listed as standard drugs for the crude drug analysis (medicinal herb analysis) in the Japanese pharmacopoeia.

### Target prediction by docking simulation

We performed a target prediction by performing a docking simulation. Protein 3D structures were obtained from the PDB database^35^ and SAHG^36^. In this study, we used AutoDock, which is a suite of automated docking tools, to predict how compounds bind to a target protein^37^. We performed a large-scale docking simulation for all possible pairs of the constituent compounds and about 40,000 human proteins. The predicted protein–ligand complexes were optimized and ranked according to the empirical scoring function, which estimates the binding free energy of the ligand receptor complex. We stored the calculated numerical results in the platform.

### Target prediction by machine learning

We performed a target prediction by using our previously developed method, called TESS (target estimation based on similarity search)^38^, to predict target proteins on the basis of compound chemical structures and large-scale chemical–protein interactome data in the framework of chemogenomics. We propose to apply the TESS algorithm to each constituent compound of Kampo medicine. In the TESS procedure, we calculated the similarity scores of compound chemical structures by the Jaccard index based on the KCF-S descriptors^34^, which were used as prediction scores.

**Figure 8:**
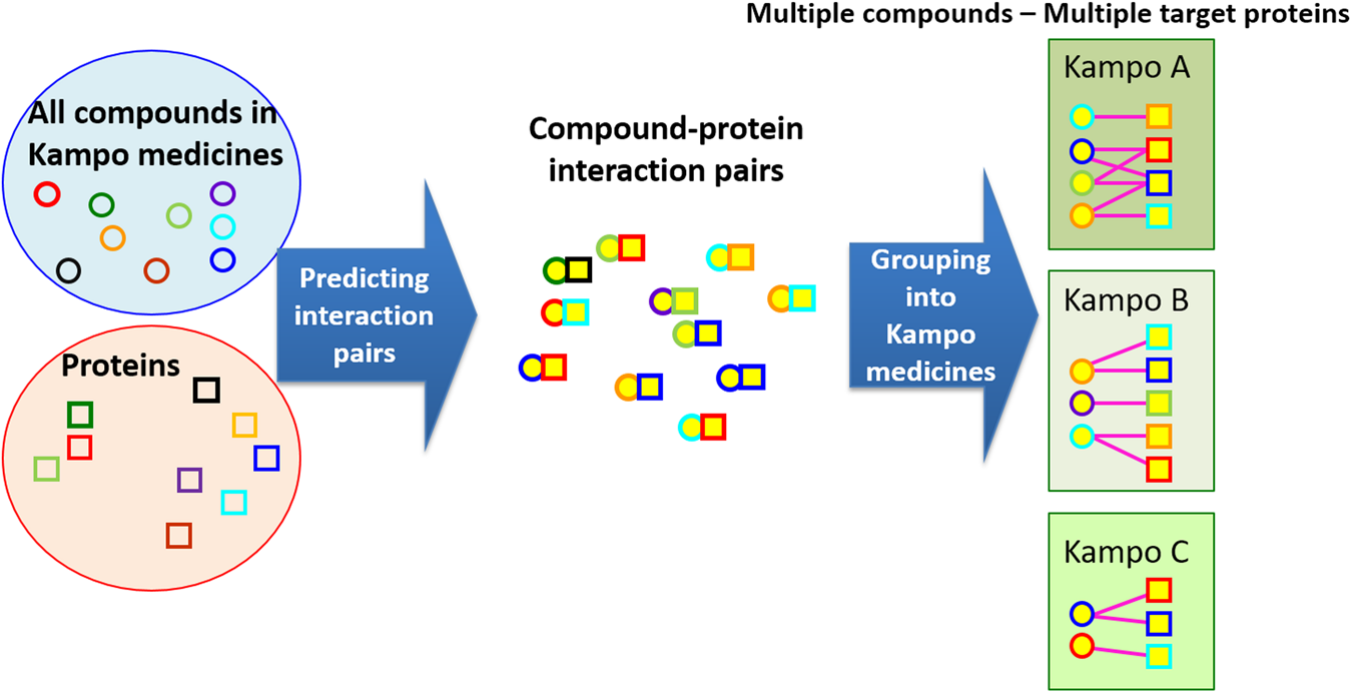
A workflow of the target perdition for Kampo medicines. Compound–protein interactions are newly predicted using compound chemical structure similarities in the framework of supervised classification. Then, the predicted compound–protein pairs are grouped into Kampo medicines based on their constituent compounds.

First, we compute pairwise similarity scores for all pairs between a query constituent compound and all of the interactome compounds in our chemical–protein interactome data. Second, from the interactome compounds known to interact with the *k*-th protein (*k* = 1, 2,…, *p*), we select an interactome compound with the highest similarity to the query constituent compound and use the corresponding similarity score as a prediction score to assess the possibility that the query compound interacts with the *k*-th protein. Third, we repeat this procedure for all *p* interactome proteins and assign the prediction scores to pairs between the query compound and all interactome proteins. Finally, high scoring compound–protein pairs are predicted as candidates for interaction pairs. Then, the predicted compound–protein pairs are grouped into Kampo medicines based on their constituent compounds. Figure 8 shows an illustration of the process. The details of the performance evaluation can be found in the “Performance evaluation” section in Supplementary Information.

### Pathway/ontology enrichment analysis

We performed the functional enrichment analyses for natural medicine (e.g., Kampo medicines, crude drugs) by mapping a set of target proteins of the constituent compounds of each natural medicine to biological pathways or molecular ontology terms. There are four options: (1) Pathway: biological pathways in KEGG PATHWAY, (2) Brite: protein classifications in KEGG BRITE, (3) Process: biological process terms in GO, and (4) Function: molecular function terms in GO. Here, we focus on the explanation of the enrichment analysis for Pathway. Note that the same procedure can be performed not only for Pathway but also for other options (Brite, Process, and Function).

We used the 163 biological pathways in KEGG (except for Global and overview maps). The enrichment ratio was calculated as the ratio of the number of associated target proteins to the number of all proteins in each pathway. The p-value was calculated by performing a hypergeometric test^39,40^. Let *G*_comp_ denote a set of target proteins of the constituent compounds of a natural medicine (e.g., Kampo medicines, crude drugs) of interest, and let *G*_path_ denote a set of target proteins in a pathway map. Further, let *r = |G*_comp_*|*, *k = |G*_path_*|*, *z = |G*_comp_ $\cap$ *G*_path_*|*, and *l* equal the total number of genes in the entire dataset (*l* = 460). We assumed that *z* follows a hypergeometric distribution. The probability of observing an intersection of size *z* between *G*_path_ and *G*_comp_ is computed as follows:

1 $$p({G}_{path},{G}_{comp})=\sum _{i=z}^{min(k,r)}(\begin{array}{c}k\\ i\end{array})(\begin{array}{c}l-k\\ r-i\end{array})/(\begin{array}{c}l\\ r\end{array})\cdot $$

The resulting p-values were corrected by using the false discovery rate^41^. In this study, Kampo medicines were associated with all possible target proteins through their constituent compounds. Several proteins were overlapped between different pathways, and the activities of protein-coding genes were not considered, rendering the enrichment analysis likely to produce high values. To determine more specific pathways, the mapping of Kampo medicines-induced gene expression data onto biological pathway maps would be a solution; however, it was out of this paper’s scope.

### Disease–target associations

The information on therapeutic target proteins for each disease was obtained from scientific literature and medical books. Drugs regulate therapeutic target proteins known to be useful for the treatment of each disease. Note that target proteins that are not known to be associated with diseases are not taken into consideration. In total, 2,062 disease–target associations involving 250 diseases and 462 therapeutic target proteins were obtained.

### Indication prediction by target matching

We performed a prediction of drug indications (i.e., applicable diseases) of the query constituent compound based on its target proteins (including known target proteins and newly predicted target proteins by TESS) and the disease–target association set.

First, we take a target protein of the query constituent compound and look for the same target protein in the disease–target association set. Second, we select diseases associated with the matched target protein, and link the query constituent compound to the selected diseases via the matched target protein. The prediction scores are set to one if the matched target proteins are known targets of the query drug, while the prediction scores are set to the TESS score if the matched target proteins are newly predicted.

### *In vivo* experiments with a CAC mouse model

Male BALB/c mice (8–10 weeks) were purchased from Japan SLC (Shizuoka, Japan). The mice were housed in the experimental animal facility at the University of Toyama and given free access to food and water. All experiments were performed in accordance with the Guide for the Care and Use of Laboratory Animals of the National Institutes of Health and the University of Toyama. The Animal Experiment Committee at the University of Toyama approved all of the animal care procedures and experiments (authorization no. A2015INM-2). CAC model was induced as described previously^42^. The mice were administered azoxymethane intraperitoneally (10 mg/kg; Sigma-Aldrich, St. Louis, MO). After 5 days, the mice were administered 2% DSS (36-50 kDa; MP Biomedicals, Santa Ana, CA) in their drinking water for 5 days, followed by 16 days of regular water. This cycle was repeated three times. The body weight of each mouse was measured every other day, and its colonic mucosa was monitored using a mouse endoscopy system (AE-C1; AVS, Tokyo, Japan). On day 70 after the start of azoxymethane administration, the mouse colon was excised for macroscopic evaluation and histological and biological analyses. Visible tumors (>1 mm along the major axis) were counted in the mid to distal colon of each mouse.

## Electronic supplementary material

Supplementary information
