# Supplementary material for: KampoDB, database of predicted targets and functional annotations of natural medicines
Source: Sci Rep. 2018 Jul 25;8:11216. doi: 10.1038/s41598-018-29516-1 (PMC6060122; doi:10.1038/s41598-018-29516-1)

SUPPLEMENTARY INFORMATION

KampoDB, database of predicted targets and functional annotations of natural medicines

Ryusuke Sawada[1], Michio Iwata[2], Masahiro Umezaki[3,6], Yoshihiko Usui[3], Toshikazu Kobayashi[3], Takaki Kubono[4], Shusaku Hayashi[4], Makoto Kadowaki[4], Yoshihiro Yamanishi[2,5,*]

[1] Medical Institute of Bioregulation, Kyushu University, 3-1-1 Maidashi, Higashi-ku, Fukuoka, Fukuoka 812-8582, Japan.
[2] Department of Bioscience and Bioinformatics, Faculty of Computer Science and Systems Engineering, Kyushu Institute of Technology, 680-4 Kawazu, Iizuka, Fukuoka 820-8502, Japan.
[3] Division of Chemo-Bioinformatics, Institute of Natural Medicine, University of Toyama, Toyama 930-0194, Japan.
[4] Division of Gastrointestinal Pathophysiology, Institute of Natural Medicine, University of Toyama, Toyama 930-0194, Japan.
[5] PRESTO, Japan Science and Technology Agency, Kawaguchi, Saitama 332-0012, Japan.
[6] Deceased.

* To whom correspondence should be addressed. Tel: +81-948-29-7821; Fax: +81-948-29-7801; Email: yamani@bio.kyutech.ac.jp

Regulation of Kampo medicines

In Japan, the Ministry of Health, Labor and Welfare (MHLW) is the regulatory agency responsible for ethical Kampo medicines. The regulations for ethical Kampo medicines as pharmaceuticals are the same as those for conventional pharmaceuticals, and are mandatory to assure the safe and effective use of Kampo products in the quality control and quality assurance aspects.

① Application Guideline for Ethical Kampo Formulation

“Application Guideline for Ethical Kampo Formulation (MHLW 1980)” indicates criteria for application and the key points to consider for each criterion. The outline of each criterion indicates the following;

- Brand name including name of Kampo formulas.
- Active ingredient's daily dose.
- Dosage, dosage interval and route of administration.
- Standards and test methods of Kampo extracts;
  - Extraction, filtration and concentration conditions, and yield.
  - Biologically active compound.

- Color, smell and form.
- Identification of individual crude drugs components.
- Impurity test: heavy metals and arsenic.

② Guideline on Data Requirements for Ethical Kampo Formulation

The quality of ethical Kampo medicines must be comparable with that of decoctions made from crude drugs in “Guideline on Data Requirements for Ethical Kampo Formulation (MHLW 1985)”.

Data should be provided on at least three batches of the drug products. Each batch must be subjected to testing at least 3 times.

③ Crude drugs and Kampo extracts in the Japanese Pharmacopoeia

The Japanese Pharmacopoeia (JP) is the official standard to assure the quality of medicines and legally binding. To assure quality of Kampo formulas, JP contains the monographs of crude drugs and Kampo medicines with high usage in Japan.

④ Good Manufacturing Practice for Kampo Products

Manufacturing of all pharmaceuticals including Kampo medicines must be done in accordance with Good Manufacturing Practice (GMP) in Japan. Since all manufacturing process in accordance with GMP is important to assure quality of Kampo medicines, a guideline for ethical Kampo medicines was published by MHLW in 1987, and “GMP for Kampo Products” was revised by MHLW in 2012.

Therefore, Kampo medicines can be identified only by their name. For example, all brands of "kakkonto" are composed of the same seven Japanese Pharmacopoeia standard medicinal herbs. Kakkonto is one of the most frequently used Kampo medicines in Japan. Although there are little differences among ratios of medicinal herb component of "kakkonto" brands within a certain limit approved by above guidelines, one brand of "kakkonto" is composed of these medicinal herbs in fixed proportions. A three-dimensional high-performance liquid chromatography profile of kakkonto provided by Tsumura & Co. (Tokyo, Japan) is shown in Supplementary Figure 1:

Performance evaluation

We evaluated the performance of the target prediction method (TESS in KampoDB) by performing the following 5-fold cross-validation experiment using constituent compounds of known target proteins (1,266 compound-protein interactions). First, we split all compounds of known target proteins into five subsets. Second, we used each subset of compounds as test compounds. Third, we

removed compounds that were identical to test compounds from the learning data in TESS. Fourth, we predicted targets of test compounds using the reconstructed learning data in TESS. Finally, we evaluated the prediction accuracy for test compounds using the Receiver Operating Characteristic (ROC) curve and Precision-Recall (PR) curve. Precision and sensitivity (recall) are dependent on a threshold of the prediction scores, so we used the two curves. Supplementary Figure 4 shows the ROC curve and PR curve. It was observed that the prediction accuracy of TESS was much higher than that of random inference.

An application to shoseiryuto

We show an application to Kampo medicine shoseiryuto. Shoseiryuto is a herbal formula of Japanese traditional kampo medicine and has been used clinically for the treatment of allergic rhinitis, bronchitis, bronchial asthma, and cold. Shoseiryuto clinically reduces the symptoms of these diseases. The pharmacological mechanism underlying the therapeutic effects of shoseiryuto in these diseases has been investigated both *in vitro* and *in vivo*. However, its mode of actions remains partially elucidated. Shoseiryuto comprises eight crude drugs: Ephedra herb, Processed ginger, Schisandra fruit, Glycyrrhiza, Pinellia tuber, Peony root, Asiasarum root, and Cassia bark.

Ephedra herb

Although the contribution of these eight crude drugs to the pharmacological effects of shoseiryuto has been partially investigated, Ephedra herb is considered to be primarily involved in the pharmacological effects of shoseiryuto for allergic rhinitis. Ephedra herb is extensively used as a nasal decongestant, bronchodilator, antitussive, and expectorant for several years, and the responsible ingredients in Ephedra herb are ephedrine and pseudoephedrine. Furthermore, the pharmacological effects of ephedrine and pseudoephedrine are primarily mediated by their adrenomimetic properties, and ephedrine works by turning on α and β adrenergic receptors.

When kampo medicine shoseiryuto is entered as a query in “Natural medicine list” of KampoDB and “Ephedra herb” is clicked, we can predict several proteins, such as α1 adrenergic receptors and β2 adrenergic receptors, as potential candidates for the target proteins of the main constituent compound ephedrine (C00001409) in Ephedra herb. When we click “hsa:148 ADRA1A,” “hsa:147 ADRA1B,” or “hsa:146 ADRA1D” among various candidates for the target proteins and browse KEGG database on α1A, α1B, or α1D adrenergic receptors, we found using the KEGG database that a target protein of oxymetazoline is α1A, α1B, and α1D adrenergic receptors. Oxymetazoline is a sympathomimetic that selectively agonizes α1 adrenergic receptors. Nowadays, oxymetazoline is available over-the-counter as a topical nasal decongestant in nasal sprays. In the nasal fossae, the regulation of the mucosal vascular network, particularly the filling and emptying of the cavernous vein plexuses, is fundamental to the regulation of the airflow and thus to the sensation of obstruction. The venous plexuses are surrounded by adrenergic nerve fibers, and they are connected to these fibers by adrenergic receptors; while β receptors are vasodilators, α receptors are vasoconstrictors and preponderant. Indeed, the application of ephedrine to the nasal mucosa in

humans decreases the nasal resistance. Selective α1 adrenergic receptor agonist oxymetazoline exerts a vasoconstrictive effect on the vessels and thus is highly effective against nasal congestion.

When we click "hsa:154 ADRB2" among several candidates for the target proteins and browse KEGG database on β2 adrenergic receptors, we found that a target protein of salbutamol is β2 adrenergic receptor. Salbutamol is a short-acting, selective β2 adrenergic receptor agonist, which is typically used for treating acute episodes of bronchospasm caused by bronchial asthma, chronic bronchitis, and other chronic bronchopulmonary disorders. In addition, β2 adrenergic receptors are crucial receptors on the bronchial smooth muscles. Notably, the activation of these receptors elevates the cAMP concentration, starting the signaling cascade that ends with the inhibition of myosin phosphorylation, lowering the intracellular concentration of calcium ions, resulting in hyperpolarization, and relaxing the bronchial smooth muscles. In addition, an increase in the cAMP concentration inhibits inflammatory cells in the airway, such as basophils, eosinophils, particularly mast cells, from releasing inflammatory mediators and cytokines. Thus, intranasal salbutamol attenuates nasal symptoms and inhibits histamine and tryptase release, thereby supporting the hypothesis that salbutamol plays a protective role in the airway by inhibiting the mast cell activation.

Processed ginger

Processed ginger has been used for various diseases, including nausea, asthma, diarrhea, cough pain, and arthritis. Processed ginger extracts are multicomponent mixtures of biologically active constituents, including the structurally related compounds gingerols, shogaols, paradol, gingerdiol, and zingerone. The medicinal properties of Processed ginger are primarily related to gingerols and shogaols. Both gingerols and shogaols exhibit anticancer, antioxidant, antimicrobial, anti-inflammatory, and antiallergic activities.

When we click "6-gingerol" and "6-shogaol" among the "List of constituent compounds" in "Target prediction" of KampoDB, we can predict candidates for the target proteins of 6-gingerol and 6-shogaol by machine learning. Among these candidates for their target proteins, the prediction scores of "transient receptor potential cation channel subfamily V member 1," "5-hydroxytryptamine receptor 1," and "arachidonate 5-lipoxygenase" are high in both simulations for 6-gingerol and 6-shogaol. Among these three candidates for their target proteins, arachidonate 5-lipoxygenase is highly involved in the pathogenesis of various diseases because it is accountable for the production of inflammatory leukotrienes, and the overproduction of leukotrienes is a primary cause of inflammation in asthma and allergic rhinitis. In addition, we found using the KEGG database that a target protein of zileuton is arachidonate 5-lipoxygenase. Zileuton, an orally active inhibitor of 5-lipoxygenase, inhibits the leukotriene formation and has been primarily used for the maintenance treatment of asthma. In addition, leukotriene-modifying agents, including zileuton, are approved by the FDA for the treatment of asthma and allergic rhinitis. Thus, we simulated the protein–ligand complex of 6-gingerol or 6-shogaol and arachidonate 5-lipoxygenase (NCBI-ProteinID: NP_000689). We perform the simulation for docking 6-gingerol or 6-shogaol to arachidonate 5-lipoxygenase and predict arachidonate 5-lipoxygenase as a target protein of 6-gingerol and 6-shogao by the docking simulation as well as machine learning.

Schisandra fruit

Schisandra fruit, which contains lignans, such as schizandrin and gomisins, as primary constituents, has been used in the treatment of gastrointestinal tract diseases, respiratory failure, and cardiovascular diseases. Some latest studies of the pharmacological properties of Schisandra fruit have reported a beneficial role in the treatment of some respiratory system disorders. The possible application of Schisandra fruit is as an anti-asthmatic drug because it lowers the airway hyperresponsiveness, immunoglobuline E level, passive cutaneous anaphylaxis reaction induced by antigen–IgE complexes, and immune cell infiltration in mice with asthma. Thus, Schisandra fruit has been used to treat chronic cough for thousands of years.

When we click “schizandrin” among the “List of constituent compounds” in “Target prediction” of KampoDB, we can predict candidates for the target proteins of “schizandrin” by the machine learning. Among these candidates for its target proteins, the prediction scores of “arachidonate 5-lipoxygenase” is high. Correspondingly, we can predict candidates for the target proteins of gomisin A and gomisin N by machine learning, and the prediction scores of arachidonate 5-lipoxygenase are high in both simulations for gomisin A and gomisin N. Thus, we simulated the protein–ligand complexes of schizandrin, 6-gingerol or 6-shogaol, and arachidonate 5-lipoxygenase (NCBI-ProteinID: NP_000689).

Glycyrrhiza

Glycyrrhiza has been used to treat various diseases from common cold to liver disease as an anti-tussive expectorant, relaxant, hepato-protective drug, and anti-inflammatory drug. Recently accumulated lines of evidence revealed that Glycyrrhiza exerts anti-inflammatory, anticancer, antioxidant, and antimicrobial effects. The biologically active components of Glycyrrhiza are liquiritin, glycyrrhizic acid, and flavones. Glycyrrhizic acid, a triterpene saponin, is a major water-soluble component of Glycyrrhiza.

When we click “Glycyrrhizic Acid” among the “List of constituent compounds” in “Target prediction” of KampoDB, we can determine candidates for the target proteins of glycyrrhizic acid by machine learning. Among the candidates for its target proteins, the prediction scores of “corticosteroid 11-beta-dehydrogenase isozyme 1 and 2” and “JUN” (KEGG Definition: transcription factor AP-1) are high. Glycyrrhizic acid is known to inhibit the conversion of “active” cortisol to “inactive” cortisone in the kidneys by inhibiting the enzyme 11-β-hydroxysteroid dehydrogenase. Consequently, cortisol accumulates within the collecting duct of the kidney, thereby causing hypertension. Thus, Glycyrrhiza should not be prescribed to patients with a known history of hypertension in doses sufficient to inhibit 11-β-hydroxysteroid dehydrogenase.

Activator protein 1 (AP-1) is a transcription factor that regulates the gene expression in response to various stimuli, including cytokines, growth factors, stress, and bacterial and viral infections. AP-1 controls numerous cellular processes, including differentiation, proliferation, and apoptosis. The AP-1 activation can act synergistically with NF-κB, resulting in the production of proinflammatory cytokines and chemokines, thereby promoting inflammatory processes. The structure of AP-1 is a heterodimer comprising proteins belonging to JUN, FOS, activating transcription factor, and JDP families. JUN participates in the macrophage activation by regulating the expression pattern of pro- and anti-inflammatory genes upon the stimulation with diverse Th1 or Th2 cytokines, pro- or anti-inflammatory stimulations, and microbial products, thereby affecting the severity of rheumatoid

arthritis. Thus, JUN acts as a checkpoint during the macrophage activation, shifting their responses toward a proinflammatory phenotype and could represent an interesting therapeutic target in rheumatoid arthritis. Thus, we simulated the protein–ligand complex of “Glycyrrhizic Acid” and “JUN” (NCBI-ProteinID: NP_002219).

In conclusion, KampoDB projects that shoseiryuto exerts a vasoconstrictive effect through α1 adrenergic receptors (Ephedra herb: ephedrine and pseudoephedrine), a relaxing effect on the bronchial smooth muscles through β2 adrenergic receptors (Ephedra herb: ephedrine and pseudoephedrine), an inhibitory effect on the mast cell activation through β2 adrenergic receptors (Ephedra herb: ephedrine and pseudoephedrine), an inhibitory effect on the leukotriene formation as a 5-lipoxygenase inhibitor (Processed ginger: 6-gingerol and 6-shogaol; Schisandra fruit: schizandrin, gomisin A and gomisin N), and anti-inflammatory effects as an AP-1 (JUN) inhibitor (Glycyrrhiza: Glycyrrhizic Acid). Overall, shoseiryuto is expected by KampoDB to effectively exhibit various therapeutic effects on allergic diseases, including bronchial asthma and allergic rhinitis.

REFERENCES

Maegawa, H., Nakamura, T. & Saito, K. Regulation of traditional herbal medicinal products in Japan.

J Ethnopharmacol **158 Pt B**, 511-515, doi:10.1016/j.jep.2014.07.012 (2014).

Supplementary Figure 1: A three-dimensional high-performance liquid chromatography profile of kakkonto provided by Tsumura & Co. (Tokyo, Japan).

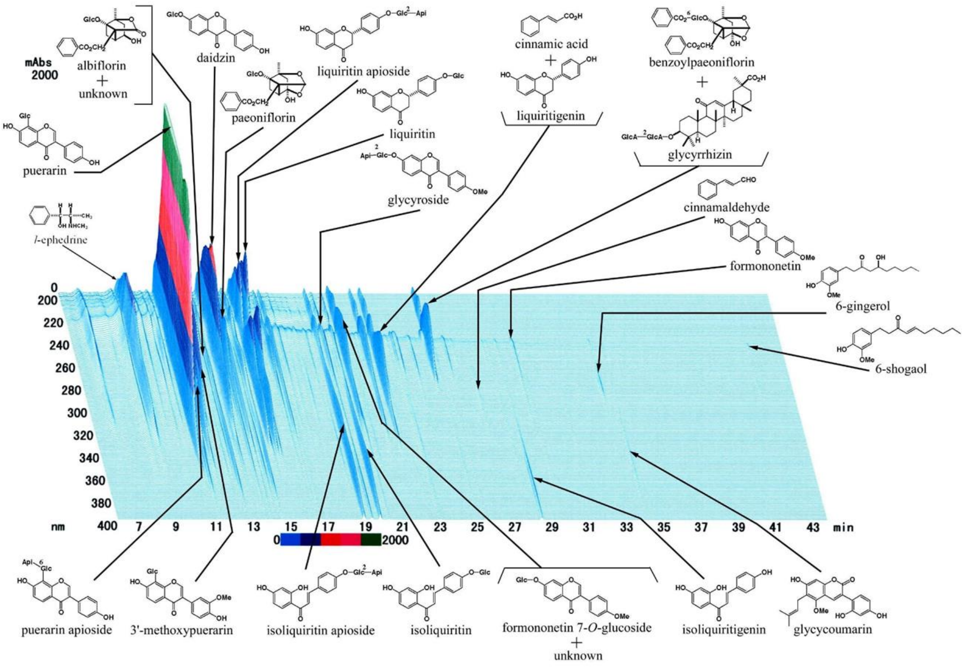

Supplementary Figure 2: A three-dimensional high-performance liquid chromatography profile of boiogito provided by Tsumura & Co. (Tokyo, Japan).

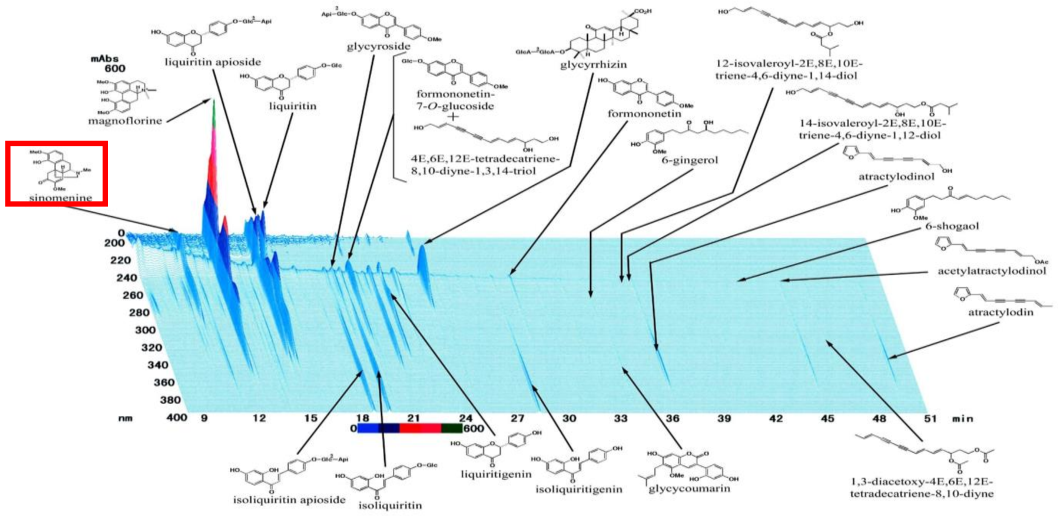

Supplementary Figure 3: A three-dimensional high-performance liquid chromatography profile of daikenchuto provided by Tsumura & Co. (Tokyo, Japan).

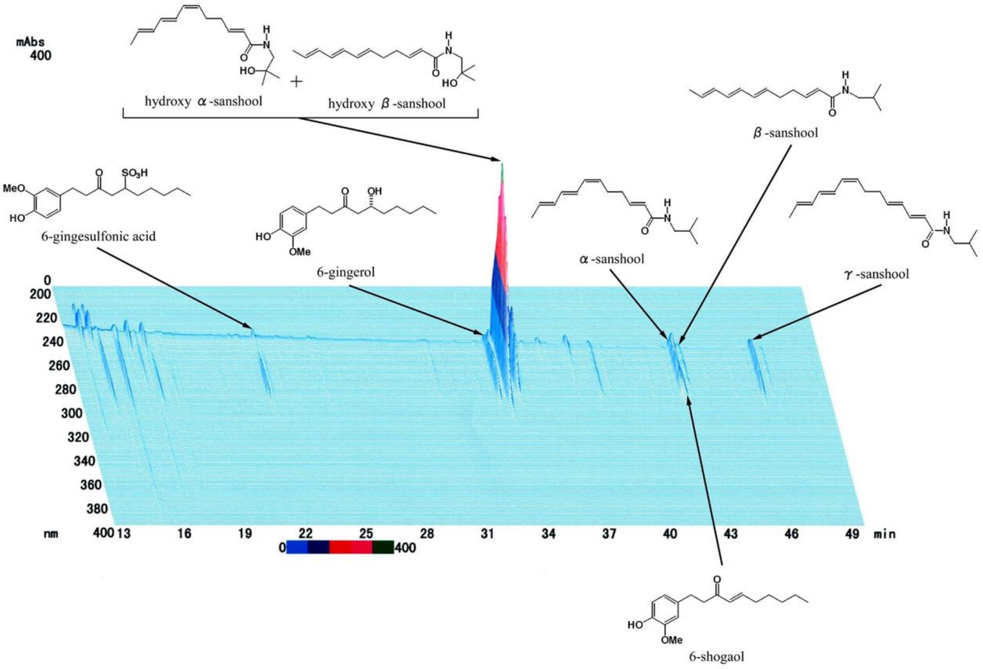

Supplementary Figure 4: Receiver Operating Characteristic (ROC) curve (A) and Precision-Recall (PR) curve (B).

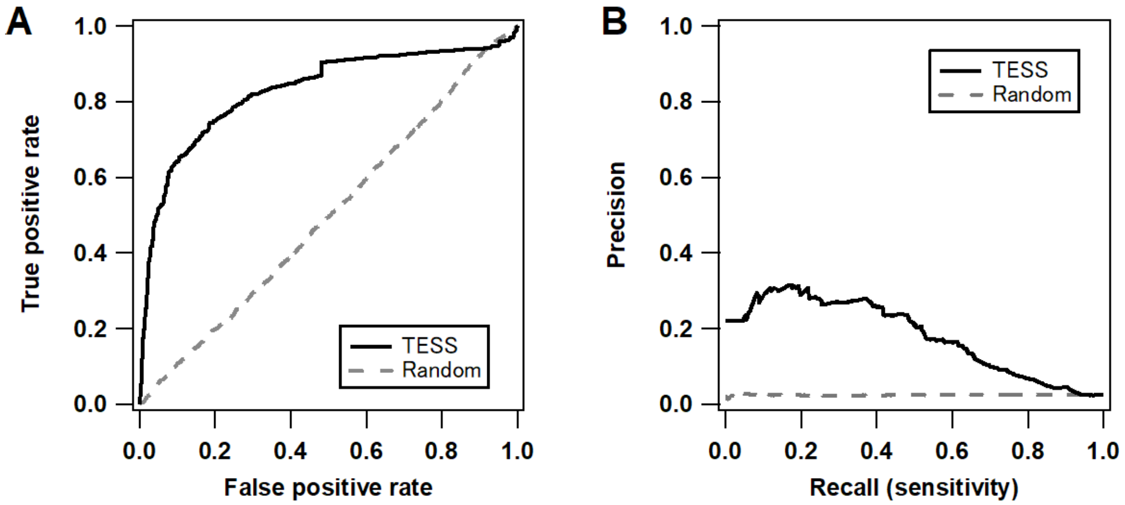

Supplement: Supplementary file 1 — Supplementary information [file 41598_2018_29516_MOESM1_ESM.pdf]
